# Primary Localized Cutaneous Nodular Amyloidosis and Limited Cutaneous Systemic Sclerosis: Additional Cases with Dermatoscopic and Histopathological Correlation of Amyloid Deposition

**DOI:** 10.3390/dermatopathology8030028

**Published:** 2021-07-02

**Authors:** Laura Atzori, Caterina Ferreli, Caterina Matucci-Cerinic, Luca Pilloni, Franco Rongioletti

**Affiliations:** 1 Dermatology Clinic, Department Medical Sciences and Public Health, University of Cagliari, 09124 Cagliari, Italy; atzoril@unica.it (L.A.); ferreli@unica.it (C.F.); rongioletti.franco@hsr.it (F.R.); 2 Clinica Pediatrica e Reumatologia, IRCCS Istituto Giannina Gaslini, DINOGMI, University of Genova, 16147 Genova, Italy; 3 Pathology Unit, Department Medical Sciences and Public Health, University of Cagliari, 09124 Cagliari, Italy; luca.pilloni@unica.it; 4 Dermatology Unit, Vita-Salute University San Raffaele, 20132 Milan, Italy

**Keywords:** primary localized cutaneous nodular amyloidosis, dermoscopy, limited cutaneous systemic sclerosis, connective tissue disease

## Abstract

Primary localized cutaneous nodular amyloidosis (PLCNA) is a rare condition due to the plasma cell proliferation and skin deposition of immunoglobulin light chains, without systemic amyloidosis or hematological dyscrasias. The association with autoimmune connective tissue diseases has been reported, especially with Sjogren’s syndrome, and in a few cases with systemic sclerosis. Herein, we describe three cases of PLCNA occurring in women with a diagnosis of limited cutaneous systemic sclerosis and review the literature on the topic to highlight a stereotypical presentation. Moreover, we support the usefulness of dermoscopy, characterized by a yellow–orange waxy pattern surrounded by telangiectasias, for a rapid and non-invasive diagnostic assessment. Thus, when asymptomatic nodules occur on lower limbs of women affected with limited systemic sclerosis, and dermoscopy identifies yellow–orange blotches, a diagnosis of PLCNA can be considered and further confirmed by histopathology. Monitoring for systemic amyloidosis development is advisable, although the risk of progression is considered very low.

## 1. Introduction

Primary localized cutaneous amyloidosis occurs when the amyloid is deposited in the skin in the absence of systemic involvement, due to the extracellular accumulation of abnormally folded proteins arranged in the beta-pleated sheet. Primary localized cutaneous amyloidosis consists of three main variants: macular amyloidosis, lichen amyloidosis and primary localized cutaneous nodular amyloidosis (PLCNA). In lichen and macular amyloidosis, amyloid is made by keratin while PLCNA is characterized by an abnormal cutaneous deposition of immunoglobulins, predominantly amyloid light-chain amyloidosis (AL), secondary to a localized proliferation of plasma cells [1,2,3,4,5]. Despite monoclonal plasma cell proliferation, no systemic hematological involvement is usually found at PLCNA diagnosis [2], although a 7% risk of myeloma development has been reported [6,7]. The occurrence of PLCNA in autoimmune diseases is well documented, especially in association with Sjögren’s syndrome [8,9,10], where nodular skin amyloidosis can also develop inside the breasts and lungs. Herein, we report three cases of PLCNA associated with limited cutaneous systemic sclerosis (lcSSc), with a review of the literature [11,12,13,14,15,16,17]. In addition to histopathology, our interest is focused on the peculiar dermoscopic characteristics that may help suspecting a diagnosis of PLCNA [18,19].

## 2. Presentation of Cases

### 2.1. Case 1

**Figure 1:**
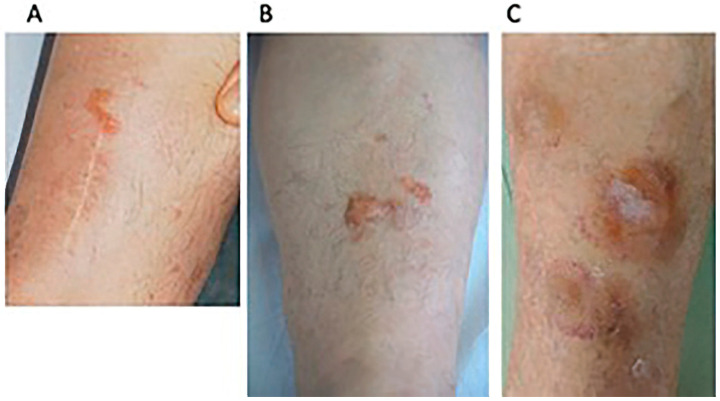
Clinical examination. (**A**) Case 1: residual well demarcated, linear orange-yellow plaque on the left leg with a white scar from a previous diagnostic biopsy; (**B**) Case 2: dome-shaped, yellow–pinkish nodule, and isolated papules of her left leg; (**C**) Case 3: multiple yellow–purple nodular lesions of the left leg.

**Figure 2:**
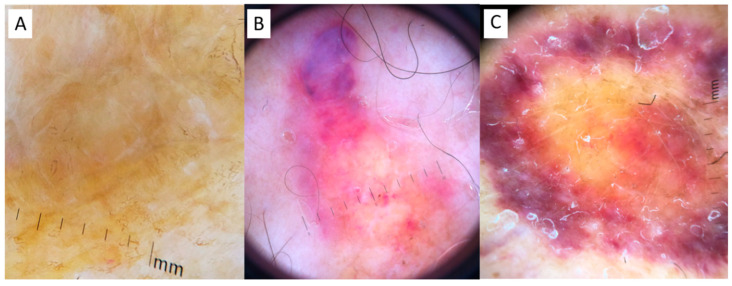
Dermoscopy. (**A**) Case 1: a structureless yellow background interspersed with whitish scar-like strikes in Case 1; (**B**) Case 2: roundish yellow waxy blotches on a hemorrhagic background, interspersed with fine telangiectasias and hemorrhagic spots; (**C**) Case 3: structureless yellow background interspersed with whitish spots, surrounded by a hemorrhagic halo with elongated serpentine vessels.

A 59-year-old woman with a decade’s history of anticentromere antibodies (ACA) positive lcSSc was referred to our clinic for the occurrence of an asymptomatic, slowly enlarging, well demarcated, orange-yellow plaque on her left leg (published elsewhere [20]). The diagnosis of PLCNA was confirmed by histopathological examination. At the time of observation (Figure 1A and Figure 2A), dermoscopy showed, under polarized light, a structureless yellow background interspersed with whitish scar-like strikes. Histopathology confirmed dermal AL deposition. Extensive work-up was negative and no signs of systemic amyloidosis were detected (This case has been previously and extensively reported [20]).

### 2.2. Case 2

**Figure 3:**
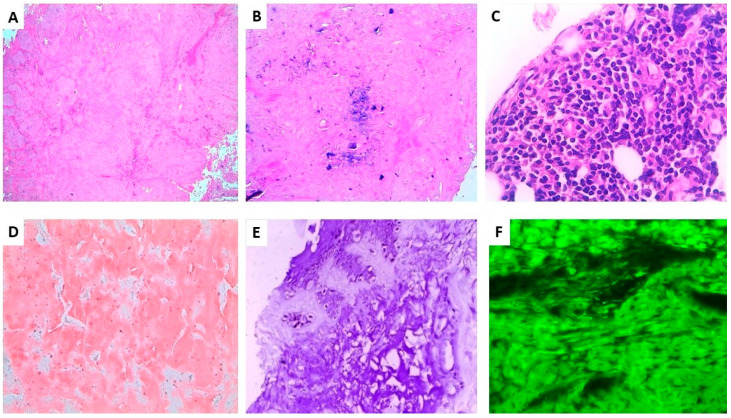
Histopathological findings of Case 2. (**A**). Nodular deposits of amorphous eosinophilic material in the dermis and subcutis (original magnification ×40) (**B**). Focal microcalcification inside the hyaline material (original magnification ×100) (**C**). Peripheral patchy focal infiltrate of lymphocytes with many plasma cells (original magnification ×400) (**D**). Amorphous material intensely stained with Congo red (original magnification ×100) (**E**). Amorphous material intensely stained with crystal violet (original magnification ×100) (**F**). Positive apple green birefringence under polarized light (original magnification ×200).

A 53-year-old ACA positive lcSSc South American woman presented with an 8-year evolving, well demarcated, dome-shaped, yellow–pinkish nodule of her left leg (Figure 1B). The clinical features were characterized by severe hand and forearm sclerosis, acral calcinosis and telangiectasias on her face. Dermoscopy showed roundish yellow waxy blotches on an hemorrhagic background surrounded and interspersed with fine telangiectasias (Figure 2B). Histopathology showed the deposition, throughout the whole dermis and subcutaneous tissue of an amorphous acellular eosinophilic material (Figure 3A) with areas of calcification (Figure 3B) and a moderate perivascular and interstitial lymphocytic and plasma cells infiltrate (Figure 3C), Congo red staining was positive on the amorphous material (Figure 3D) as well as crystal violet (Figure 3E). Positive apple green birefringence under polarized light confirmed amyloid deposition (Figure 3F). Immunohistochemistry showed a positive staining for immunoglobulin kappa chain and, with less intensity, for lambda chain. No signs of systemic amyloidosis were detected after extensive work-up.

### 2.3. Case 3

**Figure 4:**
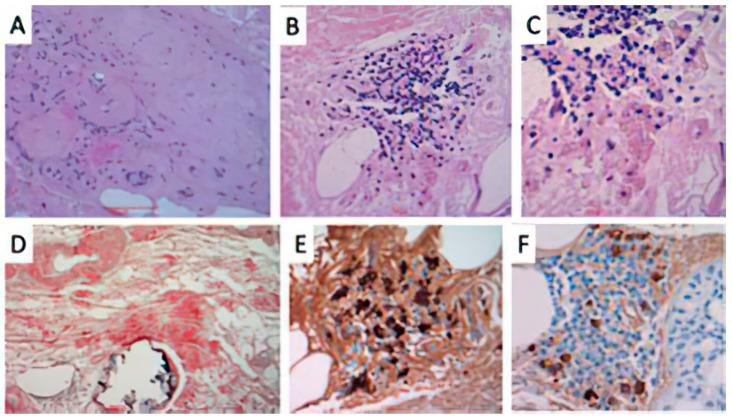
Histopathological findings of Case 3. (**A**) presence of a hyaline-like, amorphous eosinophilic material in the dermis surrounding and involving dermal vessels. (HE original staining ×40); (**B**) A perivascular and interstitial lymphocytic infiltrate (original magnification ×100) with (**C**) focal plasma cells component (original magnification ×400). (**D**) Congo red stained positively the amorphous eosinophilic deposits of amyloid in the dermis, surrounding deep dermal vessels (original magnification ×100). Immunohistochemical studies for (**E**) lambda and (**F**) kappa light chains showed evidence of lambda light-chain restriction, consistent with a monoclonal plasma cell proliferation.

A 74-year-old woman with lcSSc (ACA positive) was referred to dermatological consulting for the occurrence of bilateral well demarcated, multiple, yellow–purple nodular lesions of her legs, progressively enlarging during the last 14 months (Figure 1C). She was affected by type 2 diabetes, chronic hepatopathy, portal hypertension and a five-year disease history of recurrent digital ulcers, calcinosis, Raynaud’s phenomenon, sclerodactyly, extensive facial telangiectasias and moderate esophageal dysmotility. No treatment was supplied in consideration of the age and the general conditions. Dermoscopy of skin nodules showed, under polarized light, a structureless yellow background interspersed with whitish spots, surrounded by a hemorrhagic halo with elongated serpentine vessel (Figure 2C). Histopathology of the skin lesion showed the presence of a dermal and hypodermal homogeneous, hyaline-like amorphous eosinophilic material, involving and surrounding the thickened vessel walls (Figure 4A). The material stained positively with Congo red (Figure 4D). A slight perivascular and periadnexial histiocytic and plasmocytic infiltrate was present in the deep reticular dermis (Figure 4B,C). Immunostaining was significantly positive for lambda chains (Figure 4E) while kappa chains were barely represented in the lymphoplasmacytic infiltrate.

## 3. Discussion

The occurrence of cutaneous amyloidosis in lcSSc patients is probably more frequent than reported [11,12,13,14,15,16,17], with 12 cases of PLCNA including the present cases (Table 1). PLCNA usually follows lcSSc by several years and frequently occurs on the lower limbs of adult women affected by lcSSc.

Although PLCNA pathogenesis remains poorly understood, the autoimmune imbalance characteristic of lcSSc might play a role in the cutaneous plasma cell dysregulation. A similar explanation has been postulated for PLCNA associated with Sjögren’s syndrome [9]. However, additional local factors might explain the latency from lcSSc onset to the development of PLCNA, and the typical localization on distal legs of postmenopausal women. The progressive hardening of the soft tissues, the adnexal depletion and macro and microvascular involvement, especially on the distal extremities in SSc, might contribute to the isolation in the dermis of a pool of monoclonal plasma cells, starting to release and accumulate light chains that cannot be removed by the poor circulation. The microtraumas of these areas are another concomitant possible trigger of PLCNA, including microcalcification. In primary cutaneous amyloidosis, such as keratin-type macular and lichen amyloidosis, dermoscopy has been reported as a useful diagnostic tool, showing a white or brown central hub surrounded by reticular pigmentation, which corresponds to the histopathological findings of basal hyper pigmentation, amyloid deposition in papillary dermis and pigment incontinence [19]. In another paper, we have described the diagnostic contribution of dermoscopy in detecting PLCNA made by amyloid L [20], presenting with a different pattern characterized by yellow–orange roundish structures, furrowed by whitish hyperkeratotic strikes on an erythematous violaceous background, with more or less elongated telangiectasias [19]. This pattern of PLCNA, which has been recently confirmed by another report [21], corresponds to plasmocytic light chain-derived nodular aggregates of amyloid throughout the reticular dermis and subcutaneous tissue, infiltrating the adnexa and vessel walls with a surrounding lymphoplasmacytic infiltrate. From a dermoscopic point of view, the differential diagnosis of such yellow–orange blotches includes several histiocytic and granulomatous diseases, both inflammatory (xanthogranulomas, necrobiosis lipoidica and sarcoidosis) and infective (leishmaniasis and lupus vulgaris) [22]. The quite large structureless yellow aggregates of PLCNA are otherwise different from the small yellow dots of these granulomatous conditions, where there is a more prominent inflammatory infiltrate. However, the gold standard for diagnosis is histopathology showing throughout the dermis a nodular deposit of hyaline and eosinophilic material, also involving the walls of small vessels, the adnexa and subcutaneous tissue accompanied by an infiltrate of perivascular (monoclonal) plasma cells. Congo red staining displays the presence of a brick-red deposit in the dermis, which under polarizing microscopy shows apple–green birefringence. Positivity of the hyalin-like material with the crystal violet stain is also useful to confirm amyloid. Immunohistochemistry for serum amyloid P, although nonspecific, is positive within the eosinophilic material and immunohistochemistry showing light-chain restriction, usually lambda within the plasma cells, consistent with PLCNA. Although in PLCNA the deposition of light chain-derived amyloid is usually limited to the skin sparing internal organs, a careful follow-up is recommended for the possible evolution to systemic amyloidosis or to hematological dyscrasias, including multiple myeloma [1,7]. None of our PLCNA cases or those retrieved from the literature associated with lcSSc had such an evolution: this detail is important considering the absence of consensus recommendation on PLCNA treatment [23]. In a few cases, local intralesional triamcinolone has been reported [14], as well as surgical removal, debulking, dermo abrasion and pulsed dye laser [7,9,12,13,14], with variable results, and frequent recurrence of the nodules after any procedure. Our patients refused the therapy due to the asymptomatic nature of the lesions, the risk of scarring sequelae and the possibility of new lesion occurrence.

## 4. Conclusions

PLCNA diagnosis should be considered when cutaneous waxy non-itchy nodules occur, especially on legs of postmenopausal woman with lcSSc. The unique pattern of yellow–orange blotches on a hemorrhagic-bluish telangiectatic background is useful to address the diagnosis and suggest appropriate histopathologic assessment with special stains, which is the gold standard of diagnosis. The treatment of PLCNA is still unclear, and is currently limited to surgical excision, while patient long-term follow-up is mandatory for the risk, albeit not elevated, of the development of systemic amyloidosis.

## Figures and Tables

**Table 1:** PLCNA cases associated with scleroderma. (PLCNA primary cutaneous nodular amyloidosis; m, male; f, female,).

Age	Sex	Site of PLCNA	PLCNA Duration	Scleroderma Duration before PLCNA	Histopathological Findings	Congo Red	Ref.
61	f	Left lower leg	3 years	1 year	Amyloid extending from the superficial derma to the subcutis	+	[17]
83	f	Bilateral lower legs	25 years	Not reported	Diffuse eosinophilic aggregates of amorphous material in the superficial portion of the dermis, around vascular channels and adnexal structures with prominent plasmacells infiltrate. Deposits positive for k and lambda chains	+	[16]
61	m	Bilateral lower legs, ears	18 months	10 years	amorphous, eosinophilic periodic acid-Schiff-positive material in the papillary dermis and subcutis	+	[15]
56	f	Feet and lower legs	18 months	Not reported	Diffuse amorphous eosinophilic material surrounding adipocytes and involving vascular walls	+	[14]
71	f	Left lower leg	12 months	4 years	Scattered plasma cells and amorphous pink material in the dermis and subcutis	+	[13]
58	f	Bilateral lower legs	18 months	4 years	Amorphous pink material with perivascular accentuation and scattered plasma cells	thioflavin T +	[13]
70	f	Left lower leg	4-5 years	22 years	Amorphous pink material with admixed sparse chronic inflammation	+	[13]
60	f	Left lower leg	5 years	15 years	Dermal amorphous eosinophilic material and a background of chronic inflammation with lymphocytes and plasma cells and neovascularization. immunoglobulin light chain amyloid protein deposition	+	[12]
70	f	Upper back	Not specified	12 months	Amorphous deposits in the papillary dermis	+	[11]
62	m	Forearms	Not specified	12 months	Irregular hyperkeratosis in the epidermis and dermal sclerosis with thickened and homogeneous collagen bundles in the thickened dermis.	+	[11]
59	f	Left lower leg	3 years	10 years	See text_Case 1	+	Present report
53	f	Left lower leg	8 years	15 years	See text_Case2	+	Present report
74	f	Left lower leg	14 months	5 years	See text_Case3	+	Present report

## Data Availability

The data presented in this study are available on request from the corresponding author.
